# Direct Care Workers and Job Quality: Amplifying Voices and Identifying Empowerment Practices

**DOI:** 10.1093/geroni/igaf122.2137

**Published:** 2025-12-31

**Authors:** Jennifer Morgan, Philip Taylor, Deke Cateau

## Abstract

Direct care workers across the long-term care sector experience low compensation, few benefits (e.g. paid leave, affordable health insurance, affordable mental health coverage), and heavy workloads that leave these workers prone to both burnout and injury. These job-related challenges layer on other vulnerabilities that are keyed to social position and resource constraints. The implementation of person-centered care is dependent on the capacity and stability of direct care workers across the long-term care sector. Trends in where older adults receive care, including those living with dementia, are shifting and rely on collaboration with unpaid care partners. Direct care workers are pivotal to the ability of long-term care services and supports to deliver high quality care to older adults and people with disabilities. This symposium seeks to amplify the voices of direct care workers across the long-term care sector and identify empowerment practices that can be supported through policy and practice. The first paper, the authors provides an overview of the employment trends of direct care workers by employment and occupation. In the second paper, the authors engage a life course perspective to identify social position and relational factors that nursing assistants find relevant to their perceptions of job satisfaction and intent to stay. The third paper focuses on giving voice to direct care workers in assisted living as they characterize their work. The fourth paper identifies coping strategies and elaborates organizational policies and practices that could support direct care workers. The final paper explores these issues in the VA context.

